# Observations on the Climate, Soil, and Productions of British Guiana, &c.; with Remarks on the Diseases, Their Treatment and Prevention, Founded on a Long Experience within the Tropics

**Published:** 1836-04

**Authors:** 


Art. V.—*Observations on the Climate, Soil, and Productions of British Guiana, &c.; with Remarks on the Diseases, their Treatment and Prevention, founded on a long experience within the Tropics.* By John Hancock, m.d.—*London*, 1835, pp. 89.

There is only a very small portion of this small work which comes properly within our domain, and we are bound in justice to say that we are pleased that it is no greater. We have rarely met with a piece of medical writing more defective both in a literary and scientific point of view, than the ten pages devoted to an account of the "climate and diseases of the country;" and our regret at such a lamentable failure is enhanced by the consideration of how much might have been done by a scientific and diligent observer, with the opportunities possessed by the author. He informs us, in his preface, that "he sojourned from the year 1804 to 1828 inclusive, in South America, and chiefly in British Guiana, where he followed his professional pursuits; and with the view of acquiring some knowledge of the botany of a country most rich in medical plants, and of some peculiar practices followed with great success by the inland tribes, he frequently visited the Interior," &c. Were it not that we find appended to this pamphlet the announcement of "A Treatise on Inflammation and Fever, founded on the more successful methods pursued by certain aboriginal natives of North and South America, in the Cure of Diseases," in which it is to be presumed much of his acquired information is to be produced, we should be disposed to think, judging from the present work, that he had journeyed to little purpose, as far as medicine is concerned. He however makes some statements of facts which are deserving of notice, and which cannot be affected by illogical explanations, or obsolete pathology. He says "Tubercular consumption is unknown on the coast, and extremely rare in the mountain regions, though not unfrequent on the *Llanos*. The writer can say that he has never met with an instance of genuine tubercular phthisis on the coast of Guiana, nor a single case of calculus or stone in the bladder generated there." Dr. Hancock would seem in part to account for this exemption from such diseases, by the remarkable equability of the climate. He says "there is probably no country on the globe where the temperature is more uniform than in Guiana." He maintains that the climate is not only prophylactic, but curative of phthisis; and it is no wonder, with such opinions, that he recommends European physicians to send their consumptive patients there, in lieu of Montpelier, Naples, Rome, or Madeira.

This is certainly a matter well deserving our gravest consideration; and here again we cannot but regret that the author should have contented himself with the simple announcement of so momentous a proposition, without adducing one single fact in its support, and without supplying us with one meteorological observation illustrative of a climate capable of working such wonders. To be sure he tells us that he has "long been of opinion that the exemption from phthisis on the coast of Guiana is partly owing to the gaseous emanations from the soil, but that the main cause, as he believes, is referable to the free perspiration experienced here, together with the almost total absence of those chilling blasts which are common in other tropical regions." He gives us no insight into the nature of those "gaseous exhalations," nor does he explain why Guiana, of all the vast regions of the earth situated within the tropics, should exclusively possess this happy immunity from "chilling blasts;" nevertheless, when we recollect that, according to the statement of Dr. Clark, phthisis is most prevalent among that race (the African) of men who must constitute a large proportion of the population of Guiana, we cannot but regard the fact, announced by Dr. Hancock, of the absence, or comparative absence, of this disease from that country, as one of singular importance, and meriting the strictest examination. It is impossible for a man to be mistaken as to the existence or non-existence of such a disease as tuberculous phthisis: were the fact one of such obscurity as many pathological processes, the following statement of our author, with which we shall conclude this notice, might make us hesitate before we admitted his testimony as conclusive.

"When matter from extensive ulceration and abscess (as in the lungs) has not a free discharge, it becomes absorbed into the mass of circulating fluids, and produces an irritative fever, termed hectic. By repose, and warmth of the bed at night, the patient sweats, by which the fever abates. The sweating is an effort of nature to relieve the system of the offending humour; which is evident from this that *if we collect the clammy transudation, we find it to possess most of the properties of pus.*" P. 37.

We should like to know by what chemical or other means it can be proved, and by whom it has been proved, that the perspirations of hectic patients are purulent.

